# Combined sterno-clavicular approach as an alternative technique in hybrid exclusion of aortic arch aneurysm

**DOI:** 10.1186/1749-8090-2-36

**Published:** 2007-08-20

**Authors:** Aristotle D Protopapas, Christopher Rao, Andrew Choong, Nicholas JW Cheshire, Thanos Athanasiou

**Affiliations:** 1 Department of Biosurgery & Surgical Technology, Imperial College London, St. Mary's Hospital, London, W2 1NY, UK; 2 Academic Department of Vascular Surgery, Imperial College London, St.Mary's Hospital, London, W2 1NY, UK

## Abstract

**Background:**

We describe a modified access technique for the proximal (open) part of single stage hybrid exclusion of aneurysm of the aortic arch.

**Case presentation:**

3 patients had a bifurcated Dacron graft for the innominate and left subclavian arteries and an additional end-to-side anastomosis of the left common carotid artery on the limb to the left subclavian artery. With our modification, access to the left subclavian artery is by left subclavicular incision and creation of an anterior tunnel via the left thoracic outlet from the origin of the left subclavian artery along its anatomical course to the subclavicular plane.

**Discussion:**

Advantages and disadvantages of this technique in relation to anatomy and pathology.

## Background

A number of techniques and variations thereof are currently employed in surgery for the aortic arch.

**Figure 1:**
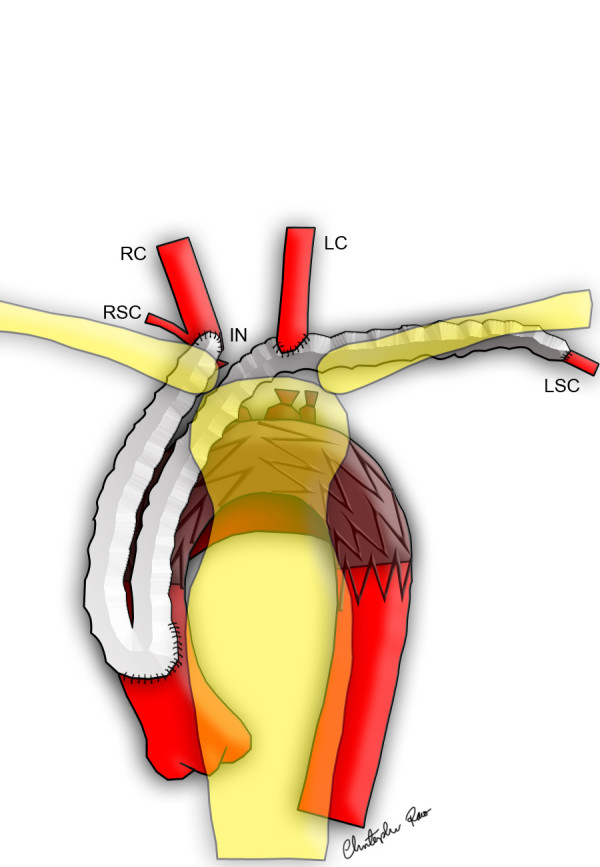
Diagram of the completed modified hybrid arch exclusion operation, note intravascular stent deployed and end-to-side graft anastomosed to left subclavian artery.

The two – [1] and single-stage [2] hybrid management of pathologies of the aortic arch have been recently described as alternatives to the traditional 'open' surgical technique with use of cardiopulmonary bypass (CPB), profound hypothermia and total circulatory arrest (TCA) [3]. Variations with the use of trifurcated grafts for arch exclusion have also been reported [4]. The primary aim of this report is to present an alternative technique of some interest: an ancillary left subclavicular incision for the left subclavian (LSC) anastomosis and use of a standard bifurcated 20 mm–10 mm Dacron graft where the left common carotid (LCC) is anastomosed end-to side (Figure 1).

## Operative Technique

Preoperative imaging with contrast thorax computed tomography is required to visualize adequately the degree of calcification of the ascending aorta and the vascular anatomy of the arch, with particular attention to the degree of calcification around the origins of head and neck vessels.

The standard preparation and draping of the patients for aortic arch surgery has been previously well described.

**Figure 2:**
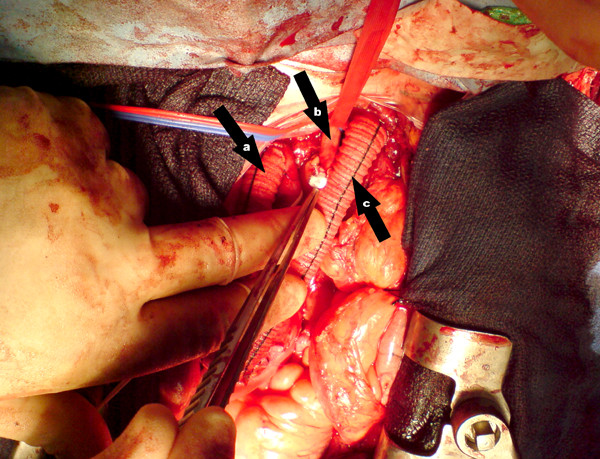
Picture of the operation in process, proximal anastomoses completed, patient in anatomical position: a: graft to Innominate Artery b: Left Common Carotid Artery c: graft to Left Subclavian Artery.

Our approach (Figure 2) specifically combines 1. extended median sternotomy with preservation of the innominate vein and 2. left subclavicular incision cephalad to the deltopectoral groove [5], transection of the left pectoralis minor muscle for access and control of the distal SCA (emphasis on preserving the trunks and divisions of the left brachial plexus).

Once proximal and distal control is achieved on the ascending aorta and the three arch vessels with colour-coded silastic slings, 5,000 international units of heparin are given intravenously. The proximal anastomosis (20 mm, 'bottom end') is fashioned following application of side-biting clamp and continuous 4.0 Polypropylene sutures were used reinforced with Teflon felt. The innominate artery (IA) anastomosis (10 mm right limb of the bifurcated graft, right top end) follows with 5.0 polypropylene suture and partial clamping of the vessel. Subsequently, whilst flow in the IA resumes, the LSC anastomosis (primary left top end) is similarly constructed after feeding the 10 mm left limb of the graft by way of a Roberts clamp and vascular tape through a tunnel leading from the sternotomy, anterior to the origin of the LSC by the thoracic outlet to the left subclavicular incision. Care is taken for the graft not to be compressed or otherwise distorted through the tunnel with temporary approximation of the sternal edges whilst fashioning the anastomosis, while important neighbouring structures (left vertebral artery, left brachial plexus, left subclavian vein and thoracic duct) are safeguarded. The proximal part of LCC is ligated with heavy silk, a vascular clamp is applied in the distal part and the LCC is divided. The last distal anastomosis (secondary left top end) is that of the distal LCC on the antero-medial aspect of the 10 mm left limb of the graft to the LSC with same suture technique and tapering.

The other two proximal parts of arch vessels (IA, LSA) are ligated with heavy silk ties to avoid endo-leak and the proximal anastomosis is marked by heavy radio-opaque clips to orientate the deployment of the endoaortic graft that is next inserted by the vascular surgeon and interventional radiologist in order to obliterate the distal part of the aneurysm. The endovascular part of the operation has also been previously well described.

With small differences in the minutiae of the operation, we have applied this technique in three patients, avoiding these with Marfan syndrome (see conclusion). The outcome was favourable in each occasion.

## Comments

Previously described advantages of hybrid technique for exclusion of aortic aneurysm are avoidance or reduction in global cerebral ischemia time, avoidance of cerebral perfusion or cerebroplegia and limitation of primary aorto-graft anastomosis to one proximal and two distal [2].

Concerns in relation to the endovascular stent exclusion have been expressed for endoleak and long-term aortic arch stability.

In the technique presented in this article two further advantages may be ascribed:

-facile access to the LSC in obese patients of short stature or barrel chest (or both) where the artery lies deep and posterior in the thoracic inlet where its approach through the sternotomy is problematic including risks of uncontrollable bleeding and injury to the left recurrent laryngeal nerve. This particular patient somatotype is where we recommend this technique.

-Optimisation of distal anastomoses when significant discrepancies exist in diameter between the limbs of Dacron grafts and head and neck vessels.

Potential disadvantages of our technique include: morbidity from the additional incision and, perhaps most importantly, compromise of the left limb of the graft by distortion in the' tunnel'. Similarly, the longer length of the left limb of the graft increases the possibility of kinking or distortion. Translocation of the LCA and injury from the side-biting clamp on the innominate are other potential risks.

We do not intend to apply this technique to patients with Marfan's somatotype to whom traction on the arch exposes adequately the proximal LSC through the sternotomy. For them, warm arch surgery with cerebral perfusion is an established alternative.

Lastly, we note the need for multidisciplinary collaboration between vascular surgeons, cardiothoracic surgeons and interventional radiologists.

## Competing interests

The author(s) declare that they have no competing interests.

## Authors' contributions

AP drafted the manuscript, CR and AC assisted in writing and creating the figures, NC and TA developed the technique and co-authored the manuscript.
